# Recent Advances in Biochemistry and Molecular Biology of Infectious Diseases

**DOI:** 10.3390/ijms24108958

**Published:** 2023-05-18

**Authors:** Salvatore Giovanni De-Simone

**Affiliations:** 1 Center for Technological Development in Health (CDTS)/National Institute of Science and Technology for Innovation in Neglected Population Diseases (INCT-IDPN), Oswaldo Cruz Foundation (FIOCRUZ), Rio de Janeiro 21040-900, RJ, Brazil; salvatore.simone@fiocruz.br; 2 Epidemiology and Molecular Systematics Laboratory (LEMS), Oswaldo Cruz Institute, Oswaldo Cruz Foundation (FIOCRUZ), Rio de Janeiro 21040-900, RJ, Brazil; 3 Program of Post-Graduation on Science and Biotechnology, Department of Molecular and Cell lar Biology, Biology Institute, Federal Fluminense University, Niterói 22040-036, RJ, Brazil

## 1. Introduction

This Editorial highlights the various observations made in the Special Issue of the *International Journal of Molecular Sciences* on “Recent Advances in Biochemistry and Molecular Biology of Infectious Diseases”. Recent epidemic outbreaks of emerging, re-emerging, and neglected infectious diseases have demonstrated the importance of applying control and prevention measures. A glaring example is the current COVID-19 pandemic caused by the severe acute respiratory syndrome coronavirus 2 (SARS-CoV-2), which has challenged researchers to understand and control the disease, leading to the development of diagnostic tests and vaccines in a very short term. Although adaptive vaccines were developed with different antigens that showed high degrees of protection, the primary focus was on the immunity induced by the spike protein, even without fully understanding the pathogenesis. The SARS-CoV-2 spreads within epithelial cells of the mucosa in the upper and, possibly, lower respiratory tracts. While virus dissemination can be controlled by the host’s immune response, a potentially lethal mechanism can arise in the second phase of the infection, diffusing to pulmonary alveoli. Marked by an uncontrolled burst of cytokines/inflammatory factors (i.e., cytokine storm), it can compromise respiratory function, and, as a consequence, lead to multi-organ failure. While mRNA-based vaccines represent the most innovative approach for prophylaxis against SARS-CoV-2-induced disease, the cumulated data indicate that the response to mRNA vaccines is ineffective in counteracting the viral replication in the upper respiratory tract, despite displaying efficacy in containing the development of severe disease. Considering that the antiviral immunity elicited by intramuscularly delivered mRNA vaccines is expected to show similar quantitative and qualitative features in the upper and lower respiratory tracts, differing outcomes are unexpected. Further investigations concluded that a possible inhibitory effect on alveolar macrophages, as a consequence of the diffusion of extracellular and/or cell-associated spike protein, can be envisioned as a key event counteracting the cytokine storm [1].

The respiratory tract is the gateway for the SARS-CoV-2 virus, and the region called the epipharynx, located behind the nasal cavity, is responsible for upper respiratory tract immunity. It is also the site of frequent acute and chronic inflammation. Previous reports have suggested that chronic epipharyngitis is involved in local symptoms such as cough and postnasal drip that are associated with systemic inflammatory diseases such as IgA nephropathy and myalgic encephalomyelitis/chronic fatigue syndrome (ME/CFS) and Long COVID. Epipharyngeal abrasive therapy (EAT), an effective treatment for chronic epipharyngitis in Japan, has been reported to be effective for these intractable diseases. The sedation of chronic epipharyngitis by EAT appears to induce the suppression of inflammatory cytokines and improve systemic symptoms, which is considered to be one of its potential mechanisms. Nishi et al. [2] compared an untreated group of individuals to patients treated with EAT to uncover the anti-inflammatory effects histologically. The expression of IL-6 in the EAT-treated group was significantly lower than in those in the EAT nontreated group (*p* = 0.0015). In addition, EAT suppressed the expression of tumor necrosis factor-alpha (TNFα), a crucial proinflammatory cytokine. As a result, continuous EAT suppressed submucosal cell aggregation and reduced inflammatory cytokines. Thus, it has been suggested that EAT may contribute to the improvement in systemic inflammatory diseases through the suppression of IL-6 expression [2].

The epipharynx is a primary target for SARS-CoV-2 replication in the early stage of COVID-19 due to its high expression of the SARS-CoV-2 entry factors angiotensin-converting enzyme 2 (ACE2) and transmembrane protease serine 2 (TMPRSS2). Nishi et al. [3] also evaluated the expression patterns of ACE2 and TMPRSS2 in tissue samples from patients before and after EAT. The results showed that the IHC scores for ACE2 and TEMPRSS2 in the EAT-treated group were 3.40-fold and 1.81-fold lower, respectively, than those in the nontreated group (*p* = 0.0208 and *p* = 0.0244, respectively). The results suggest that EAT treatment can down-regulate the expression of SARS-CoV-2 entry factors ACE2 and TMPRSS2. The authors concluded that EAT could be a potential preventative measure for COVID-19 [3].

Long COVID is the collective term used to denote the persistence of symptoms in those who have recovered from SARS-CoV-2 infection. The disease referred to as long COVID could be related to organ damage, post-viral syndrome, post-critical care syndrome, and others; thus, clinical evaluations should focus on identifying the pathophysiology followed by appropriate remedial measures. In a review by Figueres et al. [4], various aspects of long COVID including fatigue, cough, chest tightness, breathlessness, palpitations, and myalgia were analyzed with a focus on serological diagnostic usage. In people with symptoms suggestive of long COVID but without a known history of previous SARS-CoV-2 infection, serology may help to confirm the diagnosis [4].

Diagnostic testing for SARS-CoV-2 is an essential component of the global strategy for preventing and controlling COVID-19. Since the beginning of the pandemic, numerous studies have evaluated the diagnostic sensitivity of different respiratory and oral specimens for SARS-CoV-2 detection. The pandemic has since been dominated by the emergence of new variants; the latest variant being spread since November 2021 is the Omicron variant, characterized by numerous mutations and changes in host tropism in vitro that might affect the diagnostic performance of tests depending on the sampling location. In a prospective study, Figueres et al. [4] highlight the effectiveness of saliva-based RT-PCR test for the early detection of the Omicron variant.

In the same year, Kritikos et al. [5] suggested that saliva sampling might outweigh nasopharyngeal sampling when it comes to diagnosing the Omicron variant. However, in a comparative study using two different methods (Simplexa™ COVID-19 direct and Alinity mSARS-CoV-2 AMP assays), nasopharyngeal-swab (NPS) and oral saliva samples, Bordi et al. [6] found a very good inter-assay concordance (91.4 and 82.4% for saliva and NPS samples, respectively), with a significant correlation between cycle threshold (Ct) values. In addition, both platforms revealed a highly significant correlation between Ct obtained in the two matrices. Although the median Ct value was lower in NPS than in saliva samples, the Ct drop was comparable for both models after 7 days of antiviral treatment of Omicron-infected patients. Their results demonstrate that the detection of the SARS-CoV-2 Omicron variant is not influenced by the type of sample used for PCR analysis and that saliva can be used as an alternative specimen for detection. A follow-up on Omicron-infected patients could provide a new basis for further improving COVID-19 screening programs and managing patients suspected to have COVID-19.

In this issue, an article was also included that involves the development of methodologies for the determination of T epitopes of a protein from *Leishmania* spp. homologous to receptors for activated C kinase (LACK) and phosphoenolpyruvate carboxykinase (PEPCK) protein, which are strong immunogens and candidates for use in the development of novel vaccine strategies. Because *Leishmania* spp. is an intracellular protozoan with several escape mechanisms, a vaccine must provoke cellular and humoral immune responses. The study focused on the potential for T immunity based on in silico predictions and the experimental characterization of antigenic epitopes that might interact with mice or human major histocompatibility complex class I. Following predictions for immunogenicity using the Immune Epitope Database (IEDB) and the Database of MHC Ligands and Peptide Motifs (SYFPEITHI), 26 peptides were selected for interaction assays with infected mouse lymphocytes by flow cytometry and ELISpot. This strategy identified nine antigenic peptides that were strong candidates for developing a peptide vaccine against leishmaniasis [7].

Another interesting article searched for B-cell specific epitopes for diagnostic purposes using a peptide microarray methodology and describes a map of linear IgG epitopes in cholera toxin (CT) [8]. Eighteen epitopes were identified overall. Eight were identified in chain A, three in chain B, and seven in the protein P of CT. Their identification was used to develop a peptide ELISA assay for epidemiological assays.

A motivating manuscript was added, which developed a quantitative PCR (qPCR) to differentiate between infectious and inactive red sea bream iridovirus (RSIV). This qPCR assay was based on the use of propidium monoazide (PMAxx), a photoactive dye that can penetrate damaged viral particles and that binds to viral DNA. Bound viral DNA inhibits qPCR amplification, providing a mechanism to effectively differentiate infectious and inactive viruses. Furthermore, this non-invasive method will aid in establishing a disease prediction system and epidemiological analysis using seawater [9].

This issue also includes a paper that compared the classical molecular method of mechanics energy minimization with that of quantum biochemistry. The study analyzed interactions in the structure of the co-crystallized protease NS2B-NS3 of the ZIKA virus with the inhibitors benzoic acid (5YOD) and benzimidazole-1-ylmethanol (5H4I). The quantum-level results identified essential residues for stabilizing the 5YOD and 5H4I complexes after classical energy minimization, which matched previously published experimental data. These results suggest that this approach could be more promising for the design of novel inhibitors acting on NS2B-NS3 that can be extended to other pathogens [10].

*Toxoplasma gondii* is a widespread intracellular pathogen that infects humans and various animals. Huang et al. [11], studying the reposition of drugs, demonstrated that dihydroartemisinin (DHA), an effective anti-malarial drug, can also inhibit the growth of *T. gondii* with a half-maximal effective concentration (EC50) of 0.22 μM. DHA significantly increases parasites’ ROS levels and decreases the mitochondrial membrane potential, which could be reversed by ferroptosis inhibitors (DFO). Moreover, the ferroptosis inducer RSL3 inhibits *T. gondii* with an EC50 of 0.75 µM that can also enhance the DHA-induced ROS level. In combination, DHA and RSL3 significantly increased the anti-Toxoplasma effect compared to DHA alone. In summary, the paper suggests that combining DHA and RSL3 may be an alternative treatment for toxoplasmosis [11].

The study by Zhang et al. [12] focuses on the resistance to triazine components that occur during the treatment of farmed animals to intestinal coccidia (Eimeria and Cystoisospora) and provides valuable information on the gene response and mechanism of action of Eimeria to toltrazuril.

Overall, this Special Issue is a potpourri of investigative efforts applying biochemistry and molecular biology approaches to infectious disease that have generated new perspectives. These have involved (1) the identification of key events to counteract the cytokine storm that occurs in SARS-CoV-2 infection through a possible inhibitory effect on alveolar macrophages; (2) the effectiveness of saliva-based RT-PCR for the early detection of the Omicron variant; (3) EAT as a potential COVID-19 preventative method; (4) development of a methodology for the molecular diagnosis of iridovirus, and identification of T epitopes for *Leishmania* sp. for use in vaccination and B-cell specific epitopes for the development of specific serological diagnostic tests; (5) comparison of methodologies in order to trial enzymatic inhibitors, and evaluation of drug repositioning for toxoplasmosis.
